# Rapid Profiling of Volatile Organic Compounds Associated with Plant-Based Milks Versus Bovine Milk Using an Integrated PTR-ToF-MS and GC-MS Approach

**DOI:** 10.3390/molecules30040761

**Published:** 2025-02-07

**Authors:** Antonia Corvino, Iuliia Khomenko, Emanuela Betta, Federico Ivan Brigante, Luana Bontempo, Franco Biasioli, Vittorio Capozzi

**Affiliations:** 1 Research and Innovation Centre, Fondazione Edmund Mach, Via E. Mach 1, 38098 San Michele all’Adige, Italy; iuliia.khomenko@fmach.it (I.K.); emanuela.betta@fmach.it (E.B.); federico.brigante@fmach.it (F.I.B.); luana.bontempo@fmach.it (L.B.); franco.biasioli@fmach.it (F.B.); 2 Centre for Agriculture Food Environment C3A, University of Trento, Via E. Mach 1, 38098 San Michele all’Adige, Italy; 3 National Research Council of Italy, Institute of Sciences of Food Production (ISPA) c/o CS-DAT, Via Michele Protano, 71121 Foggia, Italy

**Keywords:** direct injection mass spectrometry (DIMS), volatile organic compounds (VOCs), flavour, milk, proton transfer reaction (PTR), gas chromatography (GC), mass spectrometry (MS), plant-based beverages, plant-based milks, sensory quality

## Abstract

The growing demand for plant-based beverages has underscored the importance of investigating their volatile profiles, which play a crucial role in sensory perception and consumer acceptance. This is especially true for plant-based milks (PBMs) that have a clear reference model in bovine milk. This study characterises the volatile organic compounds (VOCs) in soy, almond and oat beverages compared to bovine milk using proton transfer reaction-time of flight-mass spectrometry (PTR-ToF-MS) as a rapid and noninvasive screening tool, complemented by gas chromatography-mass spectrometry (GC-MS) for compound identification. A total of 188 mass peaks were detected by PTR-ToF-MS, all showing significant differences from the blank, while GC-MS allowed the identification of 50 compounds, supporting the tentative identifications performed with PTR-MS analysis. In order to facilitate a comparison of different milks, after statistical analysis, these 188 mass peaks were further categorised into two groups: one consisting of VOCs with minimal variability across all samples and another comprising VOCs with significantly different abundances, distinctly characterising each beverage. Principal component analysis revealed a clear separation between bovine milk and PBMs, with almond beverages exhibiting the richest volatilome, while oat beverages displayed a more homogeneous volatile profile. PTR-ToF-MS demonstrated its ability to analyse volatile profiles rapidly, with excellent complementarity to GC-MS in terms of analytical versatility. The results provided a valuable basis for testing new experimental designs aimed to characterise and enhance flavour profiles in plant-based beverages, also after processing, in case of new product development that considers using these milks as raw materials.

## 1. Introduction

The plant-based beverages market is one of the fastest-growing sectors of the food industry, with a global size of about USD 28 billion, expected to rise up to USD 73.19 billion by 2032 [1], reaching a relevant quota of the overall milk market share [2]. These trends are primarily due to a shift in attitudes among a specific population segment that is not necessarily concerned by any disease but has become more conscious about ethical and environmental issues and the importance of maintaining good health. Numerous studies have highlighted the benefits of plant-based beverages, including their potential to reduce the risk of cardiovascular diseases, improve heart health, regulate glycaemic and blood cholesterol levels, support weight management and control blood pressure [3,4]. Among plant-based beverages, plant-based milks (PBMs) represent a notable category, carefully designed to substitute and mimic the sensory and functional properties of traditional bovine milk while offering a sustainable and versatile alternative that meets evolving consumer preferences [5]. While some consumers choose PBMs as a healthy alternative to bovine milk, others are compelled to adopt them, especially those seeking a taste and texture similar to milk due to lactose intolerance or bovine milk protein allergies, conditions that may also develop over time. However, despite their perceived health benefits, it is essential to note that common ingredients in PBMs, such as nuts and soy, are known allergens. These can trigger allergic reactions ranging from mild symptoms, like hives, to severe and potentially life-threatening anaphylaxis [6]. It is important to note that PBMs, compared to bovine milk, can lack essential amino acids, vitamins and minerals [7]. Furthermore, sensory acceptability is another significant limitation to the widespread popularity of PBMs [5]. It is important to clarify that the term flavour will be used to refer to the overall sensory response to food taken into the mouth, encompassing odour, taste and other oral sensations. In contrast, taste will be used in its more specific sense, referring primarily to the chemical sensations detected by the tongue (acid, bitter, sweet or salty) and associated with nonvolatile, high molecular weight components. The terms odour, aroma and smell, which are synonymous, are primarily linked to volatile compounds, organic molecules generally characterised by a molecular weight lower than 300 Dalton, and with a considerable vapour pressure at room temperature [8]. Volatile organic compounds (VOCs) play a crucial role in determining the sensory attributes of beverages, influencing consumer acceptability and product success. The volatile profiles of beverages vary depending on the raw material, the technological treatment and the production conditions employed. Commercial PBMs often undergo conventional thermal treatments, such as ultrahigh temperature (UHT) processing, to ensure hygienic preservation and to extend product shelf-life out of the cold chain. However, these treatments can induce the development of undesirable flavours, commonly referred to as “cooked” because of lipid oxidation, degradation of amino acids, vitamins and browning reactions and other chemical changes [9,10]. This presents a substantial challenge for industries that strive to select the right raw materials, production process parameters, heat treatments and storage methods to minimise undesirable flavours, a combination of taste and olfaction [11].

Gas chromatography mass spectrometry (GC-MS) is the reference analytical technique for the quali-quantitative determination of VOCs analysis, generally requiring sample preparation for the extraction and preconcentration steps. Proton transfer reaction-time of flight-mass spectrometry (PTR-ToF-MS), on the other hand, is based on direct injection mass spectrometry (DIMS) technology and has been proven effective in broad screening and also real-time monitoring of VOCs in foods and beverages [12]. It enables multiple measurements with high sensitivity in reason of the basic sample preparation, without extraction and destruction, more compliant with the green analytical chemistry principles [13]. However, in complex food matrices, the presence of isomers and the formation of fragments and clusters can make compound identification challenging for PTR-ToF-MS. The integration of PTR-ToF-MS with GC-MS leverages the complementary strengths of both techniques, addressing their individual limitations. This combined approach enables enhanced compound identification and quantification, real-time monitoring of VOCs and dynamic volatilomics analysis, offering a powerful strategy for food and beverage research.

In PBMs, the milk alternatives sector, a general classification is not yet established; however, they can be grouped into five categories considering the raw material of origin: cereal based (oat, rice, corn, spelt); legume based (soy, peanut, lupin, cowpea); nut based (almond, coconut, hazelnut, pistachio, walnut); seed based (sesame, flax, hemp, sunflower) and pseudocereal based (quinoa, teff, amaranth) [14]. This study aims to characterise for the first time the three most popular PBMs [15] (i.e., soy, almond and oat beverages) with a rapid and noninvasive screening approach using the PTR-ToF-MS previously applied to milk [16] and serving here as a reference for animal origin products. GC-MS is employed to assist with compound identification and provide a comparative basis for the findings.

## 2. Results and Discussion

Consumers tend to prefer sustainable and naturally sweetened beverages [17], which explains the choice of organic, no-added sugar options among the analysed plant-based beverages (from the same brand), with the exception of the almond beverage containing unrefined cane sugar (see nutritional information in Materials and Methods, Section 3).

The PBMs of this study were subjected to UHT treatment to ensure hygienic preservation or roasted to stabilise the final product or increase the content of phenolic compounds and antioxidant activity [18]. However, this heat treatment can induce the development of new volatiles that may influence the flavour profile of the beverages, either positively or negatively. The undesirable flavours, commonly described as “cooked”, are the result of lipid oxidation and other chemical changes, including the degradation of amino acids, vitamins and browning reactions [9,10]. Moreover, it is important to underline that the flavour, and so the general sensory response, is also driven by the physical nature of the medium [19]. Typically, flavour potential is significantly greater in an aqueous (lipophobic) medium than in an oily (lipophilic) one. This is a behaviour that can also be influenced by the polarity of specific flavour compounds [20]. This evidence helps explain why the same VOC is perceived differently in various beverages due to its unique threshold (at parts per million levels). However, for optimal acceptance, these beverages should resemble the characteristics of animal bovine milk as closely as possible since the less a bovine milk alternative resembles dairy milk, the less likely it is to be well received, especially among consumers unaccustomed to plant-based flavours [15].

The PTR-ToF-MS technique allowed the fast and broad volatile profiling of PBMs, characterising a larger number of VOCs and VOC fragments (188 mass peaks), which was more than three times than volatiles identified by GC-MS (50 compounds).

**Figure 1:**
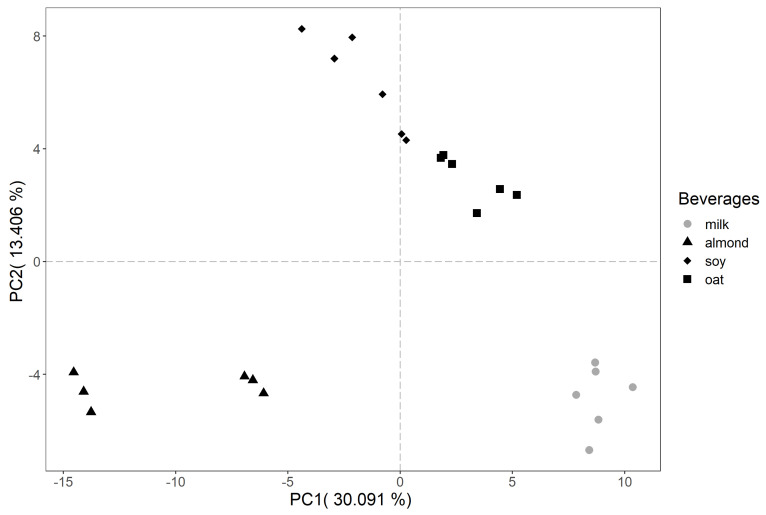
Principal component analysis (PCA) plot showing the separation of samples based on volatile organic compound (VOC) profiles from different beverages: milk (grey circles), almond (black triangles), soy (black diamonds) and oat (black squares).

**Figure 2:**
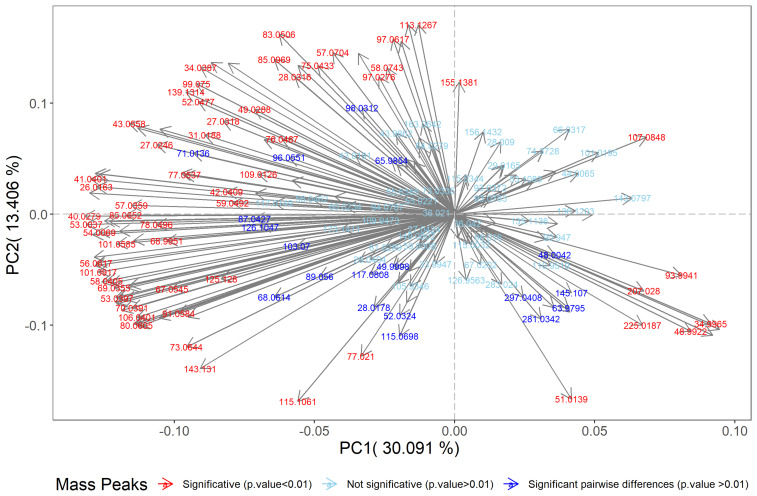
Loading plot indicating the contribution of mass peaks to the observed separation in Figure 1, with arrows representing individual VOCs. Mass peaks with significant differences among matrices (*p*-value < 0.01) are red-coloured text; mass peaks with nonsignificant or pairwise differences (*p*-value > 0.01) are coloured in shades of blue.

Multivariate data analysis was performed using principal component analysis (PCA) on the selected 188 mass peaks (one-way ANOVA, *p*-value < 0.01), with the graphical result shown in Figure 1 in order to provide a general overview of the volatile composition of four different samples of beverages analysed by PTR-ToF-MS. The first and second PCA components together explain 43% of the overall variability (PC1, 30%; PC2, 13%). On PC1, there is a clear separation between plant-based milks and bovine milk that reflects the different nutritional composition (e.g., protein, fat, sugar, minerals) that consequently influences their volatile profile [21]. The PC2 further helps in distinguishing almond beverages and bovine milk from the rest. This again confirms the distinctiveness of these two beverages, which represent the two extremes in this study based on their starting raw materials (plant-based or animal milk). The other milks (soy and oat milk-like products in our study) fall between these extremes, helping us to understand how they differ and align with consumer preference. Figure 2 represents the loading plot, which illustrates the contribution of individual volatile compounds to the observed variance and highlights their influence on the identified trends.

To further investigate the diversity in the volatile profiles of PBMs, an additional statistical analysis was performed, including ANOVA (*p*-value < 0.01) and a Fisher’s post-hoc test on the 188 mass peaks to compare the beverages. This analysis revealed two groups: (i) a group of VOCs shared across all samples or showing pairwise similarities with no significant variability and (ii) a group significantly different in terms of VOC abundance that distinctly characterised each beverage. As shown in Table 1, the most common volatiles of PBMs, with a focus on the most interesting ones, belong to the chemical classes of aldehydes, ketones, acids, alcohols, esters and furans. Terpenes and pyrazine were also detected but in low amounts. Broad insights, supported by additional studies, can be drawn regarding the various beverages, considering the general trend that the primary sensory characteristics of PBMs depend on their plant source [22].

The first group of VOCs (Table 1) provides information about the volatile compounds that quantitatively represent the common denominator among bovine and plant-based milks. It is also important to discuss VOCs that are not significantly different in terms of peak intensity to understand which volatiles contribute to the similarities among the beverages. It is interesting to delve into this first heterogeneous class of volatiles to discuss why we found these VOCs in such different beverages. Among the VOCs identified in this first group, which are common to all beverages, the *m*/*z* 117.08, with protonated formula (p.f.) C_6_H_12_O_2_H^+^ is noteworthy. This peak represents the combination of two VOCs tentatively identified (t.i.) as hexanoic acid (caproic acid) and acetic acid butyl ester (butyl acetate). Notably, this peak is more prominent in the almond beverage as this matrix contains both volatiles. Specifically, while hexanoic acid was found in all beverages, the ester was detected exclusively in the almond beverage, as confirmed by GC-MS data (Table 2). Table 2 supports this kind of comparative assessment reporting the presence of detected VOCs in the matrix under investigation based on the evidence documented in the relevant scientific literature.

Hexanoic acid has a significant impact on the flavour of many foods and a relatively high flavour perception threshold, contributing to fatty and cheesy odour [37,38]. However, in plant-based beverages, acids tend to influence taste more than aroma [14]. While caproic acid is a known component of milk fat [39], that is not the case for PBMs, where the dominant fatty acids are typically long-chain-like oleic acid (C18:1) and linoleic acid (C18:2, ω6) [40]. The presence of caproic acid in PBMs, however, raises questions. Its occurrence can be attributed to various chemical reactions, including triglyceride hydrolysis [41], lipid degradation related to the Maillard reaction [42], or through oxidation of hexanal due to the high oxidative activity observed in cereal grains, particularly oats [43]. Along with butyl acetate, other esters such as methyl 2-butoxyacetate (*m*/*z* 147.11, p.f. C_7_H_14_O_3_H^+^) and n-caproic acid vinyl ester (*m*/*z* 143.08, p.f. C_8_H_14_O_2_H^+^) were detected in all beverages. However, GC-MS analysis revealed that n-caproic acid vinyl ester was present only in almond-milk-like beverages, while methyl 2-butoxyacetate was exclusive to oat-milk-like beverages (Table 2). These findings are particularly significant as esters are highly valued for their distinctive fruity notes, which play a key role in enhancing the subtle aromatic profiles of plant-based beverages. These compounds are produced through the esterification process, involving the reaction of carboxylic acid derivatives with alcohols, and originate mainly from the oxidative breakdown of lipid precursors [14]. Another interesting VOC that can be used as a biomarker of the thermal process is oxime methoxy phenyl (*m*/*z* 152.07, p.f. C_8_H_9_NO_2_H^+^). There is limited information on oxime methoxy phenyl, a nitrogen-containing volatile, in PBMs. Oximes are formed due to a reaction between aldehydes or ketones and nitrogen-containing reducing agents in a weakly acidic medium. This reaction is often facilitated by high heat treatment and/or homogenisation. Manousi and Zachariadis [29] reported this compound in almond beverages and identified it as a dominant VOC in microfiltered pasteurised milk [44]. Additionally, it was detected in UHT milk packaged in Tetra Pak cartons [45], in ultra-pasteurised milk packaged in polyethylene terephthalate (PET) containers [46] and in reconstituted milk [47]. However, to the best of our knowledge, this is the first time oxime methoxy phenyl has been identified in oat- and soy-milk-like beverages.

On the other hand, the second group includes compounds that are, as previously mentioned, quantitatively beverage specific and provide important insights into the activated metabolic pathways. Half of the compounds had higher concentrations in the almond beverage, which exhibited a richer aromatic profile, followed by the soy beverage. The oat beverage showed a simpler aromatic profile, similar to that of bovine milk. Aldehydes and alcohols are particularly prominent in almond beverages, contributing most to the separation from the others, as confirmed by Fisher’s post-hoc test (Table 1). These two classes of compounds are generated from polyunsaturated fatty acids (e.g., linoleic acid) after the lipoxygenase enzyme action, even if it is entirely deactivated in treatment, eliminating this particular pathway of volatile formation [35]. Since almond beverage is an oil-in-water emulsion with a fat fraction high in unsaturated fatty acids, oxidation was expected to play a major role in the changes to its volatile profile. Alcohols, compared to aldehydes, have a less negative impact on the sensory attributes of food due to their high flavour thresholds [48]. The mass peak *m*/*z* 87.08, with p.f. C_5_H_10_OH^+^, groups together three alcohols t.i. as prenol (3-methyl-2-buten-1-ol), 2-penten-1-ol and 4-penten-1-ol and one ketone t.i. as 3-methyl-2-butanone. Through a reduction reaction, the alcohols are generally derived from aldehydes and ketones, two intermediate compounds, which may be transformed into other volatile compounds [49]. Prenol is considered a hemiterpenoid because it is formed by hemiterpene, consisting of a single isoprene unit (C_5_H_8_), but it is an oxygen-containing derivative and naturally present in some varieties of fruits and seeds [50]. Moreover, prenol has been reported as a constituent of an essential oil exhibiting in vitro antibacterial and anticancer activity [51]. Almonds contain many aromatic compounds and terpenoids that can be released into the beverage during extraction. Aldehydes such as hexanal (*m*/*z* 101.09, p.f. C_6_H_12_OH^+^), 2-hexenal (*m*/*z* 99.08, p.f. C_6_H_10_OH^+^), 2-heptenal (*m*/*z* 113.09, p.f. C_7_H_12_OH^+^), 2-octenal (*m*/*z* 127.11, p.f. C_8_H_14_OH^+^) and nonanal (*m*/*z* 143.13, p.f. C_9_H_18_OH^+^) are produced through lipid oxidation especially in almond beverage due to the high concentration of oleic and linoleic acids [52]. Hexanal, in particular, has been extensively studied as a reliable indicator of oxidative rancidity in foods rich in omega-6 fatty acids, such as almonds, due to its very low aroma threshold [22]. Along with nonanal, which is also statistically different from other beverages, hexanal also serves as a good indicator of the shelf life of roasted almonds [53]. As shown in Table 1, hexanal also exhibits a notable difference in soy beverages, highlighting an important aspect worth emphasising. Hexanal is a particularly significant VOC that has been studied extensively for its role in causing off-flavours in soy beverages [54]. Raw, whole soybeans contain only low concentrations of endogenous VOCs, which refers to the organic volatile compounds naturally present in raw, whole soybeans, responsible for off-flavours [55]. Endogenous VOCs in plants are formed as part of natural metabolic processes, including respiration, photosynthesis and other biochemical reactions. Some of these compounds may have positive effects, like the characteristic aromas of fruits or aromatic plants, but in some cases, as with raw soybeans, they may result in unpleasant smells or tastes. However, these compounds are rapidly generated during processing as treatments activate lipoxygenase hydroperoxide lyase pathways that convert linoleic acid to hexanal and linolenic acid to cis-3-hexenal. Unpleasant “green” and “beany” off-flavours persist by interacting with proteins in soy products, making them challenging to remove due to high affinity and rendering the product relatively unpalatable to those unaccustomed to the taste. Over the years, several methods have been proposed to mitigate these off-flavours. One approach is the heating process to inactivate lipoxygenase enzymes, as previously said; however, this can denature proteins, leading to a loss of functionality and generating additional VOCs responsible for “cooked” or “toasted” off-flavours, which are generally undesirable to consumers [56]. Preventing off-flavour formation is not easy: practical solutions are required to remove or mask them. These include flavouring additives (such as commercial cocoa- and vanilla-flavoured soy beverages) or the use of fermentation processes [25]. Interestingly, the same beany flavour of soy milk, which is often considered unpleasant by Western consumers, is actually sought after by long-time soy consumers, particularly within the Asian population, where it is strongly associated with the product’s identity and holds significant cultural importance [22]. Another aldehyde that is considered the “key flavour compound” in almonds is benzaldehyde, conferring a pleasant almond-like aroma and sweet notes, along with nonanal, which is also detected in almonds [22]. As expected, benzaldehyde was identified only in almond beverages, with the mass peak *m*/*z* 107.05 and p.f. C_7_H_6_OH^+^. It can naturally be derived from the action of β-glucosidases or be thermally generated from phenylalanine [57]. It can also originate from cinnamaldehyde, commonly found in cinnamon oil. Benzaldehyde can undergo oxidation to form benzoic acid or reduction to yield benzyl alcohol, which is also found with *m*/*z* 109.06 and p.f. C_7_H_8_OH^+^. Benzaldehyde, benzyl alcohol and some terpenes are VOCs formed during Maillard reactions [30]. VOCs are produced through these reactions, which occur between amino acids and sugars, as well as through the caramelization of sugars during thermal processing [58]. However, the contribution of Maillard reactions to volatile formation is minimal in our UHT-treated products due to their low carbohydrate content and the absence of added sugars, except for the almond beverage, which explains its more complex volatile profile.

Regarding terpenes, the *m*/*z* 155.14 t.i. as geraniol/nerol (p.f. C_10_H_1__8_OH^+^) was found in oat and soy beverages. Furans are also formed through the Maillard reaction activated by thermal processing such as cooking, roasting, baking, pasteurisation and sterilisation. In detail, the possible producing pathways are various such as thermal degradation or the rearrangement of carbohydrates alone or in the presence of amino acids, thermal degradation of some amino acids, oxidation of ascorbic acid at high temperatures and oxidation of polyunsaturated fatty acids (PUFAs) and carotenoids. The *m*/*z* 69.04 (t.i. furan and p.f. C_4_H_4_OH^+^) was found in higher concentration in almond beverage, while the *m*/*z* 97.06 (t.i. 2-ethyl furan, p.f. C_6_H_8_OH^+^) and 139.13 (t.i. 2-pentyl furan, p.f. C_9_H_14_OH^+^) were detected especially in soy beverage. The 2-pentyl furan, differently from 2-ethyl furan associated with a sweet aroma, is responsible for the unpleasant flavour, aroma and taste of soy milk. In general, the concentration of each furan is lower with respect to the other VOCs, which is very important considering the possible carcinogenic effect on humans [59]. The strategy to reduce furan levels in foods includes adjusting heating conditions but this may not always be feasible as heat is required to ensure microbiological safety. Another approach involves lowering the levels of precursors (e.g., carbohydrates, polyunsaturated fatty acids and ascorbic acid) [60]. Additionally, antioxidants such as tocopherol acetate and butyl hydroxyanisole (BHA) can be added to inhibit furan formation [61]. One additional interesting mass peak, with a strong score, is *m*/*z* 59.04, assigned as p.f. C_3_H_6_OH^+^, corresponds to acetone and appears to be primarily associated with soy beverages. Acetone is commonly found in fermented matrices, such as soy sauce, where it is identified as one of the main ketones [32]. It is also present in fermented soy milk, as noted by Yu and colleagues [62]. However, previous studies have reported acetone as a key volatile compound contributing to milk aroma [63,64], with its presence influenced by the animal’s nutritional status. Acetone levels tend to be higher in milk from cattle fed hay, wheat and silage [36].

Some generalizations can be made about the different PBMs, supported by findings from other studies. It is evident that PBMs differ significantly from bovine milk, which could explain why many consumers hesitate to try these nondairy alternatives. However, the data suggest the opposite: ethical considerations surrounding animal treatment, health consciousness and concerns about the environmental impact of meat and dairy production [65] are encouraging consumers to adapt to the flavour of PBMs. Another crucial factor for PBMs is familiarity. Research indicates that consumers tend to appreciate their unique sensory characteristics as they become more accustomed to these products instead of expecting them to mimic their animal-based counterparts [15]. Almond milk, in particular, is experiencing the highest demand, with projections for continued growth in sales [66]. In a study by Moss and colleagues [67] on consumer perceptions of plant-based milk alternatives, almond and oat beverages were found to be highly acceptable, characterised by white, fruity notes with no aftertaste and yellow, grainy, nutty, buttery and creamy qualities, respectively. In contrast, soy beverages were linked to several negative attributes, such as woody, bitter, salty, beany and grassy notes, often accompanied by a strong aftertaste [67]. This sensory profile may explain using vanillin as a masking agent to mitigate the beany and earthy flavours typical of soy-based products. These findings align with our results, where almond beverages displayed a more complex volatile profile associated with pleasant, typical VOCs. Oat beverages, on the other hand, lacked distinctive VOCs or off-flavours often found in some PBMs, exhibiting the fewest compounds in this analysis and the lowest percentage in qualitative similarity. This results in a mild, smooth taste profile, making it appealing as it avoids the beany notes associated with soy and the characteristic sweetness of almond. However, the sweetness of almond beverages may be less attractive to consumers who prefer natural sweeteners or are seeking low-calorie options. Soy beverages, while often favoured for their high protein content, are also associated with bitterness and astringency [15]. Consequently, generalizing the sensory properties of PBMs derived from different raw materials remains a challenge [14]. Therefore, variability in raw materials should be considered when producing and developing PBMs.

The ability to conduct an untargeted and rapid (with quick analysis time and no need for sample preparation, extraction or destruction) examination of VOCs associated with PBMs headspaces demonstrates the effectiveness of the PTR-ToF-MS analytical approach for fast, non-invasive quality control in the food industry [68,69]. While it has been explored in the dairy sector [70,71], its application to plant-based beverages remains underexplored.

## 3. Materials and Methods

### 3.1. Volatile Organic Compound (VOC) Determination Using Proton Transfer Reaction Time of Flight Mass Spectrometry (PTR-ToF-MS)

A multifunctional autosampler (Gerstel, Mülheim an der Ruhr, Germany) was loaded with three types of organic PBMs (oat, soy and almond beverages) from the same brand along with the reference milk of animal origin (bovine milk) purchased from a local market. The nutritional composition of these samples is detailed in Table 3.

Each sample was prepared by putting 3 mL of product into 20 mL vials and closing with a screw cap with a silicon/PTFE septum. Headspace measurements of the beverages were performed with a commercial PTR-ToF-MS 8000 apparatus from Ionicon Analytik GmbH (Innsbruck, Austria), as was already described by Capozzi et al. and Benozzi et al. [12,72]. The ionisation conditions in the drift tube involved 110 °C, 2.80 mbar drift pressure, 624 V drift voltage with an active RF mode and H_3_O^+^ as reagent ion. This led to an E/N ratio of about 140 Townsend (1 Td = 10^−17^ V  cm^2^), where E corresponds to the electric field strength and N to the gas number density. The parameter E/N influences fragmentation and clustering, making it important to work within a range of 120–140 Td [73]. The sampling time per channel of ToF acquisition was 0.1 ns, amounting to 350,000 channels for a mass spectrum ranging up to *m*/*z* = 340, which resulted in the acquisition rate of 1 spectrum/s. Each measurement was conducted automatically using a multipurpose GC automatic sampler (Gerstel GmbH, Mulheim am Ruhr, Germany) and an interval of 60 s between each measurement was applied to prevent the memory effects/carry over. The sample headspace was withdrawn with a 2.5 mL syringe (CTC Analytics AG, Zwingen, Switzerland) and injected into the static headspace (SHS) module (Ionicon Analytik GmbH, Innsbruck, Austria). The flow of zero air inside the static headspace module was 90 sccm and the syringe was injected at a speed of 100 µL/s, which provoked a 16-fold dilution of the sample. The injection time was 25 s/sample [74]. Pure N_2_ was flushed through the syringe immediately before withdrawal to prevent measurement contamination. PTR-MS performances were verified with certified calibration mixtures. Sensitivity was better than 10 cps/ppbv and the limit of detection (LOD) was lower than 100 pptv at an acquisition rate of 1 spectrum/s. The mass resolution was better than 4000 M/ΔM.

### 3.2. VOC Determination Using Gas Chromatography Mass Spectrometry (GC-MS)

GC-MS analysis was performed to help with the identification of VOCs, extracted using headspace solid phase microextraction (HS-SPME) with a 2 cm fibre coated with 50/30 μm divinylbenzene/carboxen/polydimethylsiloxane (DVB/CAR/PDMS, Supelco, Bellefonte, PA, USA). The fibre was exposed to the headspace for 45 min at 37 °C. The compounds absorbed by the SPME fibre were desorbed at 250 °C in the GC injection port for 5 min. The GC was interfaced with a mass detector operating in electron ionisation (EI) mode (internal ionisation source; 70 eV) with a scan range of *m*/*z* 33–350 (GC-MS Clarus500, PerkinElmer, Norwalk, CT, USA). Separation was carried out in an HPINNOWax fused silica capillary column (30 m, 0.32 mm ID, 0.5 µm film thickness; Agilent Technologies, Palo Alto, CA, USA). The carrier gas was helium at a constant flow rate of 2.0 mL/min. The oven temperature was programmed as follows: 40 °C (1 min)//5 °C min^−1^//250 °C (2 min). Compound identification was based on mass spectra matching with the standard NIST14 and Wiley 7th Mass Spectral Libraries and linear retention indices (LRI) were compared with the literature. LRIs were calculated under the same chromatographic conditions after injection of a C7–C30 nalkane series (Supelco, Bellefonte, PA, USA).

### 3.3. PTR-ToF-MS Spectral Data Processing and Statistical Analysis

The internal calibration of mass spectral data and peak extraction were performed according to the procedure described by [75]. Peak intensity in ppbv was estimated using the formula [73] described using a constant value for the reaction rate coefficient (k = 2 × 10^−9^ cm^3^ s^−1^). This approach introduces a systematic error for the absolute concentration of each compound that is, in most cases, below 30% and could be accounted for if the actual rate constant coefficient is available [76]. All data detected and recorded by the PTR-ToF-MS were processed using MATLAB (MathWorks, Natick, MA, USA). A total of 269 mass peaks were extracted, ranging from *m*/*z* 21 to *m*/*z* 300. A one-way analysis of variance (ANOVA) was conducted to statistically identify mass peaks significantly different (*p*-value < 0.01) from the blank samples, resulting in the identification of 188 mass peaks after removing water clusters and isotopes (see Table S1 in the Supplementary Materials). A total of 118 of these 188 mass peaks were assigned to a sum formula, whereby 48 were tentatively identified (t.i.). Tentative identification was based on GC-MS identification, the master compound assignment guide present in [77] and the literature concerning PBMs or, when not available, concerning the matrix of origin and other food categories. Before performing principal component analysis (PCA), the dataset was log-transformed to account for the expected non-normal distribution of metabolomics data. Subsequently, each variable was mean-centred and scaled to unit variance to ensure that all variables contributed equally to the analysis. PCA, analysis of variance and Fisher’s post-hoc test were performed on the final selected 188 mass peaks to spot the differences in the volatile aroma compounds emitted by the PBMs. Statistical analysis and data visualisation were performed with custom R scripts (version 4.3.2, R Foundation for Statistical Computing, Vienna, Austria) and the external packages “mixOmics”, “ggplot2” and “agricolae”.

## 4. Conclusions

The rising popularity of plant-based milks (PBMs) has spurred extensive research into methods to enhance their palatability, which remains a key challenge compared to bovine milk. One major issue is that heat treatments used to extend shelf life, such as UHT processing, often impart characteristic aromas to the products [22]. Additionally, the origin of raw materials can significantly impact the final product, especially if ingredients undergo roasting. Understanding these profiles is crucial for improving product quality and consumer acceptance. In this study, the combination of PTR-ToF-MS and GC-MS provided a synergistic approach to rapidly and effectively characterising the volatile profiles of plant-based beverages. PTR-ToF-MS does not require sample destruction for the extraction procedure, avoiding the use of solvents/toxic reagents and allowing high analysis throughput analysis, offering key insights for massive screening, real-time monitoring and compliance with the guidelines of green analytical chemistry. These features are relevant for several purposes, including the evaluation of different raw materials, technological and bio-based processes, online studies and to assure sustainable standards in R&D activities. However, GC-MS remains essential for the tentative identification of volatile organic compounds, highlighting the complementarity of the two techniques. Together, these methods offer a powerful toolset for distinguishing aromatic compounds characteristic of each sample and beverage type. Future research could focus on examining the sensory impacts of these volatiles and optimising processing conditions to enhance desirable flavours while reducing off-flavours. Expanding the analysis to include a wider variety of beverages and processing techniques may further reveal the complex interactions affecting flavour development, laying the groundwork for potential future advancements [78]. The evidence arising from this work provides a valuable basis to promote research and development activities in the field, including new product development that leverages both bovine milk and plant-based milks as raw materials.

## Figures and Tables

**Table 1:** The main volatile organic compounds (VOCs) detected through PTR-ToF-MS in each beverage were classified into two groups based on statistically significant differences determined by one-way ANOVA (*p*-value < 0.01) and Fisher’s post-hoc test. Where possible, mass peaks are assigned to protonated formulas with tentative identifications.

					Concentration (ppbv)			
Measured Mass	Theoretical Mass	Protonated Formula	Tentative Identification	*p*-Value	Almond **	Soy **	Oat **	Milk **
first group								
55.02	55.0201	C_3_H_2_OH^+^	fragment	0.42	0.56 ± 0.14 a	0.47 ± 0.13 a	0.75 ± 0.56 a	0.52 ± 0.05 a
61.03	61.0289	C_2_H_4_O_2_H^+^	acetic acid *	0.39	0.10 ± 0.01 a	0.04 ± 0.03 a	0.09 ± 0.11 a	0.06 ± 0.04 a
87.04	87.0441	C_4_H_6_O_2_H^+^	2,3 butanedione (diacetyl) *	0.16	0.14 ± 0.03 a	0.11 ± 0.03 a	0.13 ± 0.02 a	0.11 ± 0.02 b
89.06	89.0603	C_4_H_8_O_2_H^+^	3 hydroxybutanone * (acetoin)/ butanoic acid *	0.18	0.05 ± 0.01 a	0.04 ± 0.00 a	0.05 ± 0.02 a	0.04 ± 0.01 b
103.03	103.0390	C_4_H_8_O_3_H^+^	not identified	0.36	0.01 ± 0.00 a	0.02 ± 0.00 a	0.02 ± 0.00 a	0.02 ± 0.00 a
103.07	103.0759	C_5_H_10_O_2_H^+^	2 methylbutanoic acid *	0.13	0.02 ± 0.00 a	0.01 ± 0.00 a	0.02 ± 0.01 a	0.01 ± 0.00 b
109.10	109.1012	C_8_H_13_^+^	2,5-dimethylpyrazine	0.13	0.01 ± 0.00 a	0.02 ± 0.01 a	0.01 ± 0.01 b	0.01 ± 0.01 b
117.08	117.0916	C_6_H_12_O_2_H^+^	hexanoic acid */acetic acid butyl ester *	0.07	0.01 ± 0.00 a	0.01 ± 0.00 a	0.01 ± 0.00 a	0.01 ± 0.00 b
143.08	143.0703	C_8_H_14_O_2_H^+^	n-caproic acid vinyl ester *	0.27	0.00 ± 0.00 a	0.00 ± 0.00 a	0.00 ± 0.00 a	0.01 ± 0.00 a
145.11	145.1229	C_8_H_16_O_2_H^+^	octanoic acid *	0.10	0.00 ± 0.00 a	0.00 ± 0.00 a	0.00 ± 0.00 b	0.00 ± 0.00 b
147.11	147.1021	C_7_H_14_O_3_H^+^	butoxy-acetic acid methyl ester *	0.24	0.00 ± 0.00 a	0.00 ± 0.00 a	0.00 ± 0.00 a	0.00 ± 0.00 a
152.07	152.0711	C_8_H_9_NO_2_H^+^	oxime, methoxy phenyl	0.70	0.05 ± 0.01 a	0.07 ± 0.03 a	0.07 ± 0.06 a	0.06 ± 0.05 a
second group								
26.02	26.0151	C_2_H_2_^+^	alkylic fragments	2.13 × 10^−8^	0.25 ± 0.06 a	0.19 ± 0.02 b	0.10 ± 0.02 c	0.05 ± 0.02 d
27.02	27.0229	C_2_H_3_^+^	alkylic fragments	9.39 × 10^−5^	0.07 ± 0.02 a	0.07 ± 0.01 a	0.05 ± 0.01	0.03 ± 0.01 b
28.03	28.0308	C_2_H_4_^+^	alkylic fragments	2.49 × 10^−5^	0.08 ± 0.01 a	0.10 ± 0.02 b	0.07 ± 0.00 b	0.06 ± 0.01 c
31.02	31.0178	CH_2_OH^+^	formaldehyde	2.72 × 10^−9^	2.30 ± 0.31 a	2.95 ± 0.38 b	1.31 ± 0.19 c	1.41 ± 0.17 c
35.00	34.9950	H_2_SH^+^	hydrogen sulfide	2.92 × 10^−12^	0.02 ± 0.00 a	0.03 ± 0.01 b	0.02 ± 0.00 b	0.15 ± 0.03 b
39.02	39.0235	C_3_H_3_^+^	alkylic fragments	4.57 × 10^−9^	10.33 ± 2.64 a	5.02 ± 0.87 b	3.05 ± 0.75 c	1.13 ± 0.37 d
41.04	41.0386	C_3_H_5_^+^	alkylic fragment	4.51 × 10^−9^	4.26 ± 0.97 a	2.75 ± 0.34 b	1.76 ± 0.30 c	0.78 ± 0.17 d
49.01	49.0106	CH_4_SH^+^	methanethiol	2.27 × 10^−15^	0.03 ± 0.01 a	0.05 ± 0.01 b	0.05 ± 0.03 b	1.22 ± 0.19 b
51.01	51.0140	C_4_H_3_^+^	1,3-butadiyne	1.20 × 10^−9^	0.08 ± 0.01 a	0.05 ± 0.00 b	0.06 ± 0.01 c	0.11 ± 0.02 c
53.00	53.0022	C_3_OH^+^	fragment	1.44 × 10^−8^	0.76 ± 0.18 a	0.43 ± 0.06 b	0.28 ± 0.06 c	0.15 ± 0.04 d
59.05	59.0491	C_3_H_6_OH^+^	acetone *	7.95 × 10^−8^	31.04 ± 7.80 a	40.19 ± 9.71 b	6.49 ± 1.47 c	15.76 ± 3.75 d
69.04	69.0335	C_4_H_4_OH^+^	furan	1.47 × 10^−23^	0.92 ± 0.05 a	0.21 ± 0.02 b	0.10 ± 0.01 c	0.04 ± 0.01 d
69.07	69.0699	C_5_H_9_^+^	butanol	1.27 × 10^−10^	3.17 ± 0.85 a	0.44 ± 0.10 b	0.26 ± 0.08 b	0.18 ± 0.05 b
75.04	75.0441	C_3_H_6_O_2_H^+^	1 hydroxy 2 propanone (acetol) *	1.10 × 10^−8^	0.37 ± 0.04 a	0.57 ± 0.05 b	0.33 ± 0.05 b	0.31 ± 0.03 c
83.08	83.0855	C_6_H_11_^+^	3-hexen-1-ol (dehydrated form)	3.59 × 10^−11^	4.22 ± 1.05 a	0.99 ± 0.18 b	0.34 ± 0.09 c	0.08 ± 0.06 c
85.07	85.0648	C_5_H_8_OH^+^	2-pentenal/1-penten-3-one/2-methyl or 3 methyl 2 butenal	3.03 × 10^−8^	0.07 ± 0.01 a	0.04 ± 0.01 b	0.05 ± 0.01 c	0.01 ± 0.00 d
85.10	85.1017	C_6_H_13_^+^	1-hexanol (dehydrated form) *	1.95 × 10^−6^	0.22 ± 0.07 a	0.28 ± 0.04 a	0.31 ± 0.08 b	0.07 ± 0.01 c
87.08	87.0804	C_5_H_10_OH^+^	3-methyl-2-buten-1-ol * (prenol)/ 3 methyl 2 butanone */ 2 penten-1-ol */ 4 penten-2-ol *	1.60 × 10^−10^	1.64 ± 0.43 a	0.21 ± 0.05 b	0.17 ± 0.06 b	0.19 ± 0.05 b
97.06	97.0653	C_6_H_8_OH^+^	2 ethyl furan *	1.23 × 10^−15^	0.03 ± 0.00 a	0.55 ± 0.08 b	0.04 ± 0.01 b	0.01 ± 0.00 b
99.08	99.0810	C_6_H_10_OH^+^	2-hexenal *	1.23 × 10^−6^	0.05 ± 0.01 a	0.05 ± 0.01 a	0.05 ± 0.01 a	0.01 ± 0.00 b
101.09	101.0966	C_6_H_12_OH^+^	hexanal *	7.35 × 10^−11^	1.84 ± 0.48 a	0.43 ± 0.09 b	0.14 ± 0.03 b	0.04 ± 0.03 c
105.04	105.0374	C_4_H_8_OSH^+^	methional	2.51 × 10^−10^	0.08 ± 0.02 a	0.02 ± 0.00 b	0.02 ± 0.01 b	0.02 ± 0.00 b
105.08	n.a.	C_5_H_12_SH^+^/C_8_H_9_^+^	not identified	2.88 × 10^−11^	0.07 ± 0.02 a	0.01 ± 0.00 b	0.01 ± 0.00 b	0.00 ± 0.00 b
107.05	107.0491	C_7_H_6_OH^+^	benzaldehyde	3.18 × 10^−11^	2.91 ± 0.80 a	0.04 ± 0.01 b	0.02 ± 0.01 b	0.01 ± 0.01 b
107.08	107.0855	C_8_H_11_^+^	ethyl benzene	4.00 × 10^−3^	0.00 ± 0.00 a	0.01 ± 0.00 a	0.01 ± 0.01 a	0.01 ± 0.01 b
109.06	109.0648	C_7_H_8_OH^+^	benzyl alcohol	4.13 × 10^−4^	0.02 ± 0.01 a	0.00 ± 0.00 b	0.01 ± 0.00 b	0.01 ± 0.00 b
111.11	111.1174	C_8_H_15_^+^	1-octen-3-ol (dehydrated form) *	2.24 × 10^−9^	0.08 ± 0.02 a	0.03 ± 0.01 b	0.02 ± 0.00 b	0.01 ± 0.00 c
113.09	113.0966	C_7_H_12_OH^+^	2-heptenal *	3.01 × 10^−7^	0.04 ± 0.01 a	0.02 ± 0.00 b	0.01 ± 0.01 c	0.01 ± 0.01 c
113.13	113.1330	C_8_H_17_^+^	1-octanol (dehydrated form) *	4.77 × 10^−8^	0.02 ± 0.01 a	0.03 ± 0.01 a	0.04 ± 0.01 b	0.01 ± 0.00 b
115.11	115.1123	C_7_H_14_OH^+^	2 heptanone *	8.60 × 10^−9^	0.20 ± 0.06 a	0.03 ± 0.01 b	0.02 ± 0.01 c	0.14 ± 0.03 c
125.09	125.0966	C_8_H_12_OH^+^	1,5-octadien-3-one	1.72 × 10^−9^	0.03 ± 0.01 a	0.02 ± 0.00 b	0.01 ± 0.00 b	0.00 ± 0.00 c
125.13	125.1330	C_9_H_17_^+^	3-ethyl-2-methyl-1,3-hexadiene *	3.79 × 10^−3^	0.01 ± 0.00 a	0.00 ± 0.00 b	0.00 ± 0.00 b	0.00 ± 0.00 b
127.11	127.1123	C_8_H_14_OH^+^	2-octenal *	1.04 × 10^−4^	0.02 ± 0.00 a	0.02 ± 0.00 a	0.01 ± 0.00 b	0.01 ± 0.00 c
139.13	139.1123	C_9_H_14_OH^+^	2-pentyl furan *	2.25 × 10^−10^	0.06 ± 0.01 a	0.08 ± 0.02 b	0.02 ± 0.01 c	0.01 ± 0.00 d
143.13	143.1358	C_9_H_18_OH^+^	nonanal *	3.32 × 10^−9^	0.04 ± 0.01 a	0.01 ± 0.00 b	0.01 ± 0.00 c	0.02 ± 0.00 c
155.14	155.1436	C_10_H_18_OH^+^	geraniol/nerol *	5.40 × 10^−4^	0.01 ± 0.00 a	0.02 ± 0.01 a	0.02 ± 0.01 b	0.01 ± 0.00 b
169.17	169.1592	C_11_H_20_OH^+^	2-undecenal *	9.78 × 10^−8^	0.01 ± 0.00 a	0.03 ± 0.00 a	0.03 ± 0.01 b	0.00 ± 0.00 b

* VOCs also detected by SPME-GC-MS, ** soy: soy milk; oat: oat milk; almond: almond milk; milk: bovine milk. Letters were assigned based on the results of the Fisher’s post hoc test, where different letters indicate statistically significant differences between groups (*p*-value < 0.01).

**Table 2:** The main volatile organic compounds (VOCs) detected through SPME-GC-MS in each beverage were classified into chemical classes. The light grey correspondences refer to the presence of the compounds in the respective matrices. Where data are available from the scientific literature on the presence in the beverages, the references are reported; in the other cases, the association is based on the raw matrices from which the beverages are obtained.

Chemical Class	Molecular Formula	Compounds	CAS	Flavour Descriptor *	Mass	Soy **	Oat **	Alm **	Milk **
Aldehydes									
	C_6_H_10_O	2-hexenal	505-57-7	almond, sweet, apple	98,073			[23]	
	C_6_H_12_O	hexanal	66-25-1	cut grass, green	100,088	[24]		[23]	
	C_7_H_6_O	benzaldehyde	100-52-7	bitter almond, fruit, vanilla	106,041	[24,25]	[26]	[22]	[27,28]
	C_7_H_12_O	2-heptenal	18829-55-5	soap, fat	112,088	[24,25]		[29]	
	C_8_H_14_O	2-octenal	2548-87-0	fatty, green, cucumber	126,104			[30]	
	C_9_H_18_O	nonanal	124-19-6	soap, fat	142,135	[24,25]		[29]	
	C_11_H_20_O	2-undecenal	53448-07-0	fresh, fruity, orange peel, sweet	168,151			[31]	
Ketones									
	C_3_H_6_O	acetone	67-64-1	sweet, fruity, etherous	58,041	[32]		[29]	[33]
	C_3_H_6_O_2_	1-hydroxy-2-propanone (acetol)	116-09-6	sweet, caramel, ethereal	74,036				
	C_4_H_6_O_2_	2,3-butanedione (diacetyl)	431-03-8	butter, caramel, creamy	86,036				
	C_5_H_10_O	3-methyl-2-butanone	563-80-4	camphor	86,073				
	C_4_H_8_O_2_	3-hydroxybutanone (acetoin)	513-86-0	butter, creamy, green pepper	88,052		[34]		
	C_7_H_14_O	2-heptanone	110-43-0	fruity, floral, sweet, cheesy	114,104		[34]	[35]	[33]
Alcohols									
	C_5_H_10_O	2-penten-1-ol	1576-96-1	green	86,073	[24]			
	C_5_H_10_O	4-penten-2-ol	625-31-0	green	86,073				
	C_5_H_10_O	3-methyl-2-buten-1-ol (prenol)	556-82-1	fruity, green, herb, lavender	86,073				
	C_5_H_12_O	1-pentanol	71-41-0	green, wax	88,088	[24,25]	[26,34]	[22]	[33]
	C_5_H_12_O	2-methyl-1-butanol	137-32-6	ethereal, floral	88,088				
	C_5_H_12_O	3-methyl-1-butanol	123-51-3	banana, floral, fruity, malt, wheat	88,088	[22]		[22]	[36]
	C_6_H_14_O	1-hexanol	111-27-3	green, beany, fruity, grain, nutty, wheat	102,104	[22]	[26,34]	[29]	
	C_7_H_8_O	benzyl alcohol	100-51-6	balsamic, berry, floral, walnut	108,057			[29]	
	C_7_H_16_O	3,4-dimethyl-pentanol	6570-87-2		116,120				
	C_8_H_10_O	2-phenyl-ethanol	60-12-8	bitter, floral, honey, rose	122,073			[29]	
	C_8_H_16_O	1-octen-3-ol	3391-86-4	mushroom	128,120	[24,25]		[29]	
	C_8_H_18_O	1-octanol	111-87-5	bitter almond, fatty, green, rose	130,135			[29]	
Terpenes									
	C_10_H_18_O	geraniol	106-24-1	sweet, citrus and floral	154,135				
	C_10_H_18_O	nerol (cis-geraniol)	106-25-2	sweet, citrus and floral sweet rose-like odour	154,135				
Acids									
	C_2_H_4_O_2_	acetic acid	64-19-7	sour, fruity, vinegar	60,021	[25]	[22]	[22]	
	C_4_H_8_O_2_	butanoic acid	107-92-6	butter, cheese, fruit, rancid, sharp	88,052				[36]
	C_5_H_10_O_2_	2-methyl-butanoic acid	116-53-0	acid, cheese, pungent, sour	102,068				
	C_3_H_7_NO_3_	2-aminooxypropanoic acid	2786-22-3		105,042				
	C_6_H_12_O_2_	hexanoic acid	142-62-1	fatty, cheesy	116,083			[35]	[36]
	C_8_H_16_O_2_	octanoic acid	124-07-2	cheese, fatty, oily	144,115				[36]
Esters									
	C_6_H_12_O_2_	acetic acid butyl ester	123-86-4	banana, ethereal, fruity, pear	116,083				
	C_8_H_14_O_2_	n-caproic acid vinyl ester	3050-69-9	fruity	142,099				
	C_7_H_14_O_3_	butoxy-acetic acid methyl ester	10228-54-3		146,094				
Furans									
	C_6_H_8_O	2-ethyl furan	3208-16-0	burnt, earthy, malty, sweet	96,057				
	C_9_H_14_O	2-pentyl furan	3777-69-3	beany off-flavours	138,104			[23]	
Others									
	C_7_H_14_	1,1-dimethylcyclopentane	1638-26-2		122,109				
	C_9_H_16_	3-ethyl-2-methyl-1,3-hexadiene	61142-36-7		124,125				
	C_9_H_18_	2,4 dimethyl-1-heptene	19549-87-2		126,140				
	C_8_H_9_NO_2_	oxime, methoxy- phenyl	67160-14-9		151,063			[29]	[29]
	C_13_H_28_	tridecane	629-50-5	alkane	184,219				[33]

* flavour descriptor from https://foodb.ca/ (accessed on 3 January 2025), ** soy: soy milk; oat: oat milk; alm: almond milk; milk: bovine milk.

**Table 3:** Nutritional values and ingredients per 100 mL of beverages tested in this study, as reported in the product label.

Product	List of Ingredients	Energy	Total Fat	SFAs *	Total Carbohydrate	Sugars	Fiber	Protein	Salt	Calcium	Vitamin E
almond drink	water, unrefined cane sugar, almond 2.8%, rice starch, sea salt	111 kJ/27 kcal	2.5 g	0.2 g	0 g	0 g	0.6 g	0.8 g	0.1 g		1 mg
soy drink	water, Italian soy seeds 8%	178 kJ/ 43 kcal	2.6 g	0.6 g	0.7 g	0 g	0.5 g	3.9 g	0.03 g		
oat drink	water, Italian oat 11%, cold-pressed sunflower oil, sea salt	187 kJ/45 kcal	1.5 g	0.3 g	7 g	4 g	0.5 g	0.5 g	0.09 g		
milk	bovine milk	195 kJ/46 kcal	1.6 g	1.1 g	4.8 g	4.8 g		3.2 g	0.13 g	120 mg	

* SFAs = saturated fatty acids.

## Data Availability

The datasets generated for this study are available upon request to the corresponding authors.

## Supplementary Materials

The following supporting information can be downloaded at: https://www.mdpi.com/article/10.3390/molecules30040761/s1, Table S1: Volatile organic compounds (VOCs) selected after one-way analysis of variance (ANOVA) to statistically identify mass peaks significantly different (*p*-value < 0.01) from the blank samples, resulting in the identification of 188 mass peaks after removing water clusters and isotopes.
