# Supplementary material for: Rapid Profiling of Volatile Organic Compounds Associated with Plant-Based Milks Versus Bovine Milk Using an Integrated PTR-ToF-MS and GC-MS Approach
Source: Molecules. 2025 Feb 7;30(4):761. doi: 10.3390/molecules30040761 (PMC11858441; doi:10.3390/molecules30040761)
Supplement: Supplementary file 1 [file molecules-30-00761-s001.zip › molecules-3440701-supplementary.pdf]

Article

Rapid Profiling of Volatile Organic Compounds Associated with Plant-Based Milks *versus* Bovine Milk by an Integrated PTR-ToF-MS and GC-MS Approach

Antonia Corvino [1,2,*], Iuliia Khomenko [1], Emanuela Betta [1], Federico Ivan Brigante [1], Luana Bontempo [1], Franco Biasioli [1], Vittorio Capozzi [3,*]

[1] Research and Innovation Centre, Fondazione Edmund Mach, via E. Mach 1, 38098 San Michele all'Adige (TN), Italy; antonia.corvino@fmach.it, iuliia.khomenko@fmach.it, emanuela.betta@fmach.it, Federico.brigante@fmach.it, luana.bontempo@fmach.it, franco.biasioli@fmach.it
[2] Centre for Agriculture Food Environment C3A, University of Trento, San Michele All'Adige, Trento, Italy; antonia.corvino@unitn.it
[3] National Research Council of Italy Institute of Sciences of Food Production (ISPA) c/o CSDAT, via Michele Protano, 71121 Foggia, Italy; vittorio.capozzi@cnr.it

* Correspondence: vittorio.capozzi@cnr.it,Vittorio Capozzi; antonia.corvino@fmach.it, Antonia Corvino

Table S2. Volatile organic compounds (VOCs) selected after one-way analysis of variance (ANOVA) to statistically identify mass peaks significantly different (*p-value* < 0.01) from the blank samples, resulting in the identification of 188 mass peaks after removing water clusters and isotopes.

Measured mass	Theoretical mass	Protonated formula	Almond*			Soy*			Oat*			Milk*		
26.02	26.0163	C2H2+	0.25	±	0.06	0.19	±	0.02	0.10	±	0.02	0.05	±	0.02
27.02	27.0246	C2H3+	0.07	±	0.02	0.07	±	0.01	0.05	±	0.01	0.03	±	0.01
27.03	27.0318		0.04	±	0.01	0.04	±	0.01	0.02	±	0.01	0.01	±	0.01
27.04	27.0424		0.03	±	0.00	0.02	±	0.01	0.03	±	0.01	0.02	±	0.01
28.01	28.009		2.41	±	0.06	2.61	±	0.27	2.63	±	0.38	2.52	±	0.19
28.02	28.0178	CH2N+	0.16	±	0.05	0.08	±	0.06	0.11	±	0.09	0.12	±	0.03
28.03	28.0316	C2H4+	0.08	±	0.01	0.10	±	0.02	0.07	±	0.00	0.06	±	0.01
29.02	29.0165		66.10	±	1.10	68.32	±	3.81	68.63	±	5.11	67.78	±	2.84
29.04	29.0404	C2H5+	0.67	±	0.13	0.58	±	0.04	0.67	±	0.11	0.62	±	0.16
31.02	31.0188	CH3O+	2.30	±	0.31	2.95	±	0.38	1.31	±	0.19	1.41	±	0.17
33.99	33.9947	O2+	11.48	±	0.12	11.41	±	0.11	11.37	±	0.25	11.44	±	0.13
34.04	34.0387		0.33	±	0.06	0.51	±	0.11	0.23	±	0.04	0.07	±	0.02

35.00	34.9965	H2SH+	0.02	±	0.00	0.03	±	0.01	0.02	±	0.00	0.15	±	0.03
35.04	35.0392		0.06	±	0.01	0.09	±	0.02	0.05	±	0.01	0.02	±	0.00
36.02	36.021		1.26	±	0.04	1.26	±	0.08	1.24	±	0.07	1.24	±	0.05
36.04	36.042		0.05	±	0.01	0.05	±	0.00	0.05	±	0.02	0.05	±	0.01
39.02	39.0227	C3H3+	10.33	±	2.64	5.02	±	0.87	3.05	±	0.75	1.13	±	0.37
40.03	40.0279		0.49	±	0.12	0.29	±	0.04	0.19	±	0.03	0.08	±	0.02
41.04	41.0401	C3H5+	4.26	±	0.97	2.75	±	0.34	1.76	±	0.30	0.78	±	0.17
42.01	42.0115	N3	0.51	±	0.11	0.40	±	0.07	0.23	±	0.04	0.11	±	0.03
42.03	42.0271		0.00	±	0.00	0.00	±	0.01	0.01	±	0.01	0.10	±	0.02
42.04	42.0409		0.31	±	0.04	0.37	±	0.04	0.09	±	0.01	0.19	±	0.05
43.02	43.0191	C2H3O+	2.56	±	0.62	2.96	±	0.76	3.41	±	4.25	1.91	±	1.57
43.03	43.0294	CH3N2	0.64	±	0.14	1.05	±	0.22	0.48	±	0.06	0.17	±	0.06
43.06	43.0558	C3H7+	1.15	±	0.22	1.18	±	0.10	0.65	±	0.12	0.27	±	0.09
43.99	43.9862		0.02	±	0.01	0.02	±	0.01	0.02	±	0.00	0.02	±	0.01
44.01	44.0065		0.37	±	0.01	0.39	±	0.03	0.40	±	0.03	0.39	±	0.03
44.03	44.0262	H2N3	0.35	±	0.06	0.22	±	0.04	0.14	±	0.08	0.09	±	0.04
44.06	44.0609		0.03	±	0.01	0.03	±	0.01	0.02	±	0.01	0.01	±	0.00
45.99	45.9909	NO2	1.36	±	0.10	1.53	±	0.37	1.63	±	0.47	1.51	±	0.27
48.00	48.0042		0.24	±	0.01	0.23	±	0.02	0.26	±	0.04	0.26	±	0.02
48.99	48.9922	HO3+	0.00	±	0.00	0.00	±	0.00	0.01	±	0.00	0.03	±	0.01
49.01	49.0121	CH5S+	0.03	±	0.01	0.05	±	0.01	0.05	±	0.03	1.22	±	0.19
49.03	49.0288	CH5O2+	0.05	±	0.01	0.07	±	0.01	0.04	±	0.00	0.04	±	0.01
50.00	49.9998	C3N	3.21	±	0.05	3.17	±	0.11	3.06	±	0.16	3.16	±	0.09
51.01	51.0139	C4H3+	0.08	±	0.01	0.05	±	0.00	0.06	±	0.01	0.11	±	0.02
51.03	51.0292		0.10	±	0.03	0.09	±	0.03	0.06	±	0.02	0.01	±	0.01
52.00	52.0043		0.02	±	0.00	0.02	±	0.00	0.02	±	0.00	0.02	±	0.01
52.03	52.0324		0.03	±	0.01	0.03	±	0.01	0.03	±	0.01	0.03	±	0.00
52.05	52.0477		0.02	±	0.01	0.02	±	0.01	0.01	±	0.01	0.00	±	0.00
53.00	53.0037	C3HO+	0.76	±	0.18	0.43	±	0.06	0.28	±	0.06	0.15	±	0.04

53.04	53.0397	C4H5+	0.78	±	0.12	0.32	±	0.02	0.24	±	0.07	0.22	±	0.04
53.11	53.1083		0.00	±	0.00	0.00	±	0.00	0.00	±	0.00	0.00	±	0.00
54.01	54.0069		0.03	±	0.01	0.02	±	0.00	0.01	±	0.00	0.01	±	0.00
54.04	54.0441		0.04	±	0.01	0.02	±	0.00	0.01	±	0.00	0.01	±	0.00
55.02	55.0221	C3H3O+	0.56	±	0.14	0.47	±	0.13	0.75	±	0.56	0.52	±	0.05
55.07	55.0679		1.37	±	0.43	0.16	±	0.09	0.07	±	0.08	0.00	±	0.01
55.64	55.6385		0.00	±	0.00	0.00	±	0.00	0.00	±	0.00	0.00	±	0.00
55.93	55.9346		0.07	±	0.01	0.07	±	0.00	0.07	±	0.01	0.07	±	0.01
56.05	56.0462		0.13	±	0.03	0.10	±	0.01	0.13	±	0.06	0.10	±	0.01
56.06	56.0617		0.22	±	0.04	0.08	±	0.01	0.03	±	0.01	0.01	±	0.01
57.04	57.0359	C3H5O+	0.73	±	0.12	0.53	±	0.05	0.52	±	0.19	0.35	±	0.04
57.07	57.0704	C4H9+	1.23	±	0.23	1.69	±	0.16	2.27	±	0.38	0.58	±	0.11
57.94	57.9358		0.04	±	0.00	0.05	±	0.00	0.04	±	0.00	0.05	±	0.00
58.04	58.0405	CH4N3+	0.13	±	0.03	0.06	±	0.01	0.03	±	0.01	0.03	±	0.01
58.07	58.0743		0.06	±	0.01	0.07	±	0.01	0.11	±	0.02	0.03	±	0.01
59.05	59.0492	C3H7O+	31.04	±	7.80	40.19	±	9.71	6.49	±	1.47	15.76	±	3.75
59.93	59.9308		0.02	±	0.00	0.02	±	0.00	0.02	±	0.01	0.02	±	0.00
59.97	59.9677		0.00	±	0.00	0.01	±	0.01	0.01	±	0.00	0.01	±	0.00
60.02	60.0234		0.02	±	0.01	0.02	±	0.01	0.02	±	0.00	0.02	±	0.01
61.03	62.019	C2H5O2+	0.10	±	0.01	0.04	±	0.03	0.09	±	0.11	0.06	±	0.04
63.98	63.9795		0.03	±	0.01	0.03	±	0.01	0.03	±	0.01	0.04	±	0.00
64.03	64.0279		0.02	±	0.01	0.03	±	0.02	0.02	±	0.01	0.02	±	0.01
65.99	65.9854		0.00	±	0.00	0.00	±	0.00	0.00	±	0.00	0.00	±	0.00
66.03	66.0317		0.00	±	0.00	0.01	±	0.00	0.01	±	0.00	0.01	±	0.00
67.02	67.0202	C4H3O+	0.01	±	0.00	0.00	±	0.00	0.00	±	0.00	0.01	±	0.00
67.05	67.0545	C5H7+	0.18	±	0.03	0.08	±	0.02	0.07	±	0.07	0.06	±	0.05
68.03	68.0287		0.03	±	0.00	0.01	±	0.00	0.00	±	0.00	0.00	±	0.00
68.06	68.0614		0.02	±	0.00	0.01	±	0.00	0.01	±	0.01	0.01	±	0.01
69.00	68.9951	C3HO2+	0.02	±	0.00	0.01	±	0.00	0.01	±	0.00	0.01	±	0.00

69.04	69.0355	C4H5O+	0.92	±	0.05	0.21	±	0.02	0.10	±	0.01	0.04	±	0.01
69.07	69.0699	C5H9+	3.17	±	0.85	0.44	±	0.10	0.26	±	0.08	0.18	±	0.05
70.04	70.0391		0.05	±	0.01	0.01	±	0.00	0.01	±	0.00	0.00	±	0.00
70.07	70.0746		0.19	±	0.05	0.04	±	0.01	0.03	±	0.01	0.02	±	0.01
71.01	71.0136	C3H3O2+	0.02	±	0.00	0.02	±	0.00	0.02	±	0.01	0.02	±	0.00
71.05	71.0503	C4H7O+	0.08	±	0.01	0.05	±	0.01	0.05	±	0.01	0.04	±	0.01
71.08	71.0848	C5H11+	0.97	±	0.26	0.74	±	0.07	0.44	±	0.11	0.20	±	0.09
73.03	73.0335		0.14	±	0.00	0.12	±	0.01	0.17	±	0.10	0.12	±	0.01
73.06	73.0644	C4H9O+	1.52	±	0.39	0.46	±	0.12	0.55	±	0.17	0.66	±	0.19
74.37	74.3728		0.00	±	0.00	0.00	±	0.00	0.00	±	0.00	0.00	±	0.00
75.04	75.0433	C3H7O2+	0.37	±	0.04	0.57	±	0.05	0.33	±	0.05	0.31	±	0.03
76.05	76.0467		0.02	±	0.00	0.03	±	0.00	0.02	±	0.00	0.02	±	0.00
77.02	77.021		0.07	±	0.01	0.05	±	0.01	0.06	±	0.01	0.06	±	0.01
77.05	77.0537		0.27	±	0.06	0.31	±	0.06	0.08	±	0.02	0.14	±	0.03
78.05	78.0496		0.02	±	0.01	0.02	±	0.01	0.01	±	0.00	0.01	±	0.00
80.06	80.0605		0.13	±	0.03	0.01	±	0.00	0.01	±	0.01	0.01	±	0.01
83.05	83.0506	C5H7O+	0.13	±	0.01	0.19	±	0.02	0.17	±	0.02	0.04	±	0.01
83.08	83.0826	C6H11+	4.22	±	1.05	0.99	±	0.18	0.34	±	0.09	0.08	±	0.06
84.05	84.0531		0.01	±	0.00	0.02	±	0.00	0.01	±	0.00	0.01	±	0.00
85.03	85.0267	C4H5O2+	0.03	±	0.00	0.04	±	0.01	0.04	±	0.02	0.03	±	0.01
85.07	85.0652	C5H9O+	0.07	±	0.01	0.04	±	0.01	0.05	±	0.01	0.01	±	0.00
85.10	85.0969	C6H13+	0.22	±	0.07	0.28	±	0.04	0.31	±	0.08	0.07	±	0.01
87.04	87.0427	C4H7O2+	0.14	±	0.03	0.11	±	0.03	0.13	±	0.02	0.11	±	0.02
87.08	87.0788	C5H11O+	1.64	±	0.43	0.21	±	0.05	0.17	±	0.06	0.19	±	0.05
89.06	89.056	C4H9O2+	0.05	±	0.01	0.04	±	0.00	0.05	±	0.02	0.04	±	0.01
90.95	90.947		0.14	±	0.01	0.15	±	0.01	0.14	±	0.01	0.15	±	0.01
91.06	91.0584	C4H11S+	0.07	±	0.01	0.05	±	0.00	0.05	±	0.01	0.05	±	0.01
93.04	93.0372	C3H9OS+	0.98	±	0.17	1.08	±	0.07	0.97	±	0.06	1.05	±	0.07
93.08	93.0783		0.00	±	0.00	0.00	±	0.00	0.00	±	0.01	0.00	±	0.00

93.95	93.9515		0.05	±	0.00	0.05	±	0.01	0.05	±	0.00	0.05	±	0.00
93.99	93.9941		0.00	±	0.00	0.00	±	0.00	0.00	±	0.00	0.01	±	0.00
94.08	94.0787		0.01	±	0.00	0.01	±	0.00	0.01	±	0.01	0.01	±	0.01
96.02	96.016		0.02	±	0.00	0.02	±	0.00	0.02	±	0.00	0.02	±	0.00
96.06	96.0551		0.01	±	0.00	0.02	±	0.01	0.01	±	0.00	0.01	±	0.01
96.09	96.0894		0.01	±	0.00	0.01	±	0.00	0.01	±	0.01	0.01	±	0.01
97.03	97.0276	C5H5O2+	0.04	±	0.01	0.07	±	0.01	0.05	±	0.01	0.04	±	0.01
97.06	97.0617	C6H9O+	0.03	±	0.00	0.55	±	0.08	0.04	±	0.01	0.01	±	0.00
97.10	97.0994	C7H13+	0.24	±	0.06	0.07	±	0.01	0.03	±	0.00	0.02	±	0.00
98.03	98.0312		0.01	±	0.00	0.01	±	0.00	0.01	±	0.00	0.01	±	0.00
98.07	98.0668		0.01	±	0.00	0.04	±	0.01	0.00	±	0.00	0.00	±	0.00
98.10	98.1033		0.03	±	0.01	0.01	±	0.00	0.01	±	0.00	0.00	±	0.00
99.00	99.0048		0.10	±	0.02	0.11	±	0.03	0.11	±	0.03	0.11	±	0.02
99.04	99.0373		0.01	±	0.00	0.03	±	0.01	0.01	±	0.00	0.01	±	0.00
99.08	99.075	C6H11O+	0.05	±	0.01	0.05	±	0.01	0.05	±	0.01	0.01	±	0.00
100.08	100.0762		0.02	±	0.00	0.02	±	0.01	0.02	±	0.00	0.02	±	0.00
101.02	101.0195		0.02	±	0.00	0.03	±	0.00	0.03	±	0.00	0.03	±	0.01
101.06	101.0585	C5H9O2+	0.06	±	0.01	0.03	±	0.01	0.03	±	0.01	0.02	±	0.00
101.09	101.0917	C6H13O+	1.84	±	0.48	0.43	±	0.09	0.14	±	0.03	0.04	±	0.03
103.03	103.0324	C4H9O3+	0.01	±	0.00	0.02	±	0.00	0.02	±	0.00	0.02	±	0.00
103.07	103.07	C5H11O2+	0.02	±	0.00	0.01	±	0.00	0.02	±	0.01	0.01	±	0.00
105.04	105.0418	C4H9OS+	0.08	±	0.02	0.02	±	0.00	0.02	±	0.01	0.02	±	0.00
105.08	105.076	C5H13S+ / C8H9+	0.07	±	0.02	0.01	±	0.00	0.01	±	0.00	0.00	±	0.00
105.93	105.9346		0.02	±	0.00	0.02	±	0.00	0.02	±	0.00	0.02	±	0.00
106.04	106.0401	C3H8NO3+	0.03	±	0.01	0.00	±	0.00	0.00	±	0.00	0.00	±	0.00
107.05	107.0462	C7H7O+	2.91	±	0.80	0.04	±	0.01	0.02	±	0.01	0.01	±	0.01
107.08	107.0848	C8H11+	0.00	±	0.00	0.01	±	0.00	0.01	±	0.01	0.01	±	0.01
107.95	107.9492		0.03	±	0.01	0.03	±	0.00	0.03	±	0.00	0.03	±	0.00
108.95	108.9497		0.14	±	0.01	0.13	±	0.01	0.13	±	0.01	0.13	±	0.01

109.01	109.0126		0.01	±	0.00	0.01	±	0.00	0.01	±	0.00	0.01	±	0.00
109.06	109.0647	C7H9O+	0.02	±	0.01	0.00	±	0.00	0.01	±	0.00	0.01	±	0.00
109.10	109.1005	C8H13+	0.01	±	0.00	0.02	±	0.01	0.01	±	0.01	0.01	±	0.01
109.95	109.9473		0.01	±	0.00	0.01	±	0.00	0.01	±	0.00	0.01	±	0.00
110.03	110.0253		0.00	±	0.00	0.00	±	0.00	0.00	±	0.00	0.00	±	0.00
110.95	110.9519		0.01	±	0.00	0.01	±	0.00	0.01	±	0.00	0.01	±	0.00
110.10	110.104		0.00	±	0.00	0.00	±	0.00	0.00	±	0.00	0.00	±	0.00
111.04	111.0394		0.06	±	0.01	0.07	±	0.00	0.06	±	0.02	0.06	±	0.01
111.08	111.0788	C7H11O+	0.02	±	0.00	0.01	±	0.00	0.01	±	0.00	0.00	±	0.00
111.11	111.1124	C8H15+	0.08	±	0.02	0.03	±	0.01	0.02	±	0.00	0.01	±	0.00
113.02	113.0207		0.04	±	0.01	0.04	±	0.00	0.04	±	0.01	0.04	±	0.01
113.09	113.093	C7H13O+	0.04	±	0.01	0.02	±	0.00	0.01	±	0.01	0.01	±	0.01
113.13	113.1267	C8H17+	0.02	±	0.01	0.03	±	0.01	0.04	±	0.01	0.01	±	0.00
115.00	115.0032		0.01	±	0.00	0.01	±	0.00	0.01	±	0.00	0.01	±	0.00
115.03	115.0344		0.01	±	0.00	0.01	±	0.00	0.01	±	0.01	0.01	±	0.00
115.07	115.0698		0.01	±	0.00	0.01	±	0.00	0.01	±	0.00	0.01	±	0.01
115.11	115.1061	C7H15O+	0.20	±	0.06	0.03	±	0.01	0.02	±	0.01	0.14	±	0.03
117.01	117.0149		0.01	±	0.00	0.01	±	0.00	0.01	±	0.00	0.01	±	0.00
117.08	117.0808	C6H13O2+	0.01	±	0.00	0.01	±	0.00	0.01	±	0.00	0.01	±	0.00
119.09	119.0854	C9H11+	0.10	±	0.01	0.04	±	0.00	0.02	±	0.01	0.02	±	0.01
121.10	121.099	C9H13+	0.01	±	0.00	0.01	±	0.00	0.01	±	0.01	0.01	±	0.01
121.95	121.9492		0.00	±	0.00	0.00	±	0.00	0.00	±	0.00	0.00	±	0.00
123.94	123.9356		0.02	±	0.00	0.02	±	0.00	0.02	±	0.00	0.02	±	0.00
125.03	125.0339		0.03	±	0.00	0.01	±	0.00	0.01	±	0.00	0.01	±	0.00
125.09	125.0904	C8H13O+	0.03	±	0.01	0.02	±	0.00	0.01	±	0.00	0.00	±	0.00
125.13	125.128	C9H17+	0.01	±	0.00	0.00	±	0.00	0.00	±	0.00	0.00	±	0.00
125.95	125.9451		0.04	±	0.00	0.04	±	0.00	0.04	±	0.01	0.04	±	0.00
126.10	126.1047		0.00	±	0.00	0.00	±	0.00	0.00	±	0.00	0.00	±	0.00
126.90	126.8966		0.01	±	0.00	0.01	±	0.00	0.01	±	0.00	0.01	±	0.00

126.96	126.9563		0.01	±	0.00	0.01	±	0.00	0.01	±	0.00	0.02	±	0.00
127.11	127.1084	C8H15O+	0.02	±	0.00	0.02	±	0.00	0.01	±	0.00	0.01	±	0.00
133.10	133.0977		0.01	±	0.00	0.00	±	0.00	0.00	±	0.00	0.01	±	0.00
135.11	135.1136		0.01	±	0.00	0.01	±	0.00	0.01	±	0.02	0.01	±	0.01
136.12	136.1203		0.00	±	0.00	0.00	±	0.00	0.00	±	0.01	0.00	±	0.00
138.13	138.1306		0.01	±	0.00	0.02	±	0.01	0.04	±	0.06	0.04	±	0.05
139.13	139.1314	C9H14OH+	0.06	±	0.01	0.08	±	0.02	0.02	±	0.01	0.01	±	0.00
143.08	143.0797	C8H15O2+	0.00	±	0.00	0.00	±	0.00	0.00	±	0.00	0.01	±	0.00
143.13	143.131	C9H18O+	0.04	±	0.01	0.01	±	0.00	0.01	±	0.00	0.02	±	0.00
144.90	144.9015		0.02	±	0.00	0.02	±	0.00	0.02	±	0.00	0.02	±	0.00
145.11	145.107	C8H17O2+	0.00	±	0.00	0.00	±	0.00	0.00	±	0.00	0.00	±	0.00
147.11	147.1101	C7H15O3+	0.00	±	0.00	0.00	±	0.00	0.00	±	0.00	0.00	±	0.00
149.06	149.0602	C9H9O2+	0.01	±	0.00	0.01	±	0.00	0.01	±	0.00	0.01	±	0.00
151.11	151.1114	C10H15O+	0.00	±	0.00	0.00	±	0.00	0.00	±	0.00	0.00	±	0.00
152.07	152.0711	C8H9NO2H+	0.00	±	0.00	0.01	±	0.00	0.01	±	0.01	0.01	±	0.00
155.14	155.1381	C10H19O+	0.01	±	0.00	0.02	±	0.01	0.02	±	0.01	0.01	±	0.00
156.14	156.1432		0.00	±	0.00	0.00	±	0.00	0.00	±	0.00	0.00	±	0.00
157.14	157.143		0.01	±	0.00	0.01	±	0.00	0.01	±	0.00	0.01	±	0.00
163.06	163.0642		0.00	±	0.00	0.00	±	0.00	0.00	±	0.00	0.00	±	0.00
167.13	167.1274		0.00	±	0.00	0.00	±	0.00	0.00	±	0.00	0.00	±	0.00
169.17	169.1665	C11H21O+	0.01	±	0.00	0.03	±	0.00	0.03	±	0.01	0.00	±	0.00
173.14	173.1411		0.00	±	0.00	0.00	±	0.00	0.00	±	0.00	0.00	±	0.00
203.92	203.9218		2.85	±	0.16	2.53	±	0.56	2.45	±	0.62	2.58	±	0.45
204.93	204.9269		0.16	±	0.01	0.15	±	0.03	0.14	±	0.03	0.15	±	0.02
207.03	207.028		0.01	±	0.00	0.01	±	0.00	0.01	±	0.00	0.01	±	0.00
223.04	223.0424		0.01	±	0.00	0.01	±	0.00	0.01	±	0.00	0.02	±	0.01
225.02	225.0187		0.01	±	0.00	0.01	±	0.00	0.01	±	0.00	0.02	±	0.01
281.03	281.0342		0.02	±	0.00	0.02	±	0.01	0.02	±	0.01	0.03	±	0.02
283.02	283.024		0.00	±	0.00	0.00	±	0.00	0.00	±	0.00	0.01	±	0.00

297.04	297.0408	0.01	±	0.00	0.01	±	0.00	0.01	±	0.00	0.01	±	0.01
299.02	299.0166	0.01	±	0.00	0.01	±	0.00	0.01	±	0.00	0.01	±	0.01

** Soy: soy milk; oat: oat milk; alm: almond milk; milk: bovine milk.*
